# Non-host Resistance: DNA Damage Is Associated with SA Signaling for Induction of PR Genes and Contributes to the Growth Suppression of a Pea Pathogen on Pea Endocarp Tissue

**DOI:** 10.3389/fpls.2017.00446

**Published:** 2017-04-04

**Authors:** Lee A. Hadwiger, Kiwamu Tanaka

**Affiliations:** Department of Plant Pathology, Washington State University, PullmanWA, USA

**Keywords:** non-host resistance, DNA damage, salicylic acid, PR genes, *Fusarium solani*

## Abstract

Salicylic acid (SA) has been reported to induce plant defense responses. The transcriptions of defense genes that are responsible for a given plant’s resistance to an array of plant pathogens are activated in a process called non-host resistance. Biotic signals capable of carrying out the activation of pathogenesis-related (PR) genes in pea tissue include fungal DNase and chitosan, two components released from *Fusarium solani* spores that are known to target host DNA. Recent reports indicate that SA also has a physical affinity for DNA. Here, we report that SA-induced reactive oxygen species release results in fragment alterations in pea nuclear DNA and cytologically detectable diameter and structural changes in the pea host nuclei. Additionally, we examine the subsequent SA-related increase of resistance to the true pea pathogen *F. solani* f.sp. *pisi* and the accumulation of the phytoalexin pisatin. This is the first report showing that SA-induced PR gene activation may be attributed to the host pea genomic DNA damage and that at certain concentrations, SA can be temporally associated with subsequent increases in the defense response of this legume.

## Introduction

The salicylic acid (SA) signal receptor protein NPR1 has been reported in *Arabidopsis* (Wu et al., 2012), and NPR1 is a known link between SA signaling and defense gene activation. An alternate hypothesis for signal reception in the legume, pea, indicates that host cell chromatin can both serve as a receptor (Hadwiger, 2015a) and provide the site for increased transcription of pathogenesis-related (PR) genes (Isaac et al., 2009). DNA damage within chromatin can also initiate signaling cascades in animal tissues (De Dieuleveult et al., 2016) and is dependent on ubiquitin (Stewart et al., 2009). In rice and peas, chromatin changes can result in the suppression of innate immunity (Li et al., 2015) or the enhancement of PR gene transcription (Chang et al., 1995; Hadwiger, 2009, 2015b; Isaac et al., 2009), respectively. Recent reports (Neaualt et al., 1996; Yan et al., 2013) indicate that SA has an affinity for DNA, suggesting the potential of a DNA target site for SA that may add to or supersede reception by a cytoplasmic protein. The model pea endocarp/bean pathogen interaction system is a suitable system to research the role of SA in non-host defense in legumes.

Non-host resistance differs as it is more durable than the single dominant resistance genes commonly manipulated by plant breeders. However, both mechanisms are associated with the enhanced synthesis of PR proteins that are usually involved in plant defense (Klosterman et al., 2001). Genes for many of the PR proteins have been cloned (van Loon, 1985), and their antifungal properties have been identified, e.g., PR2, β-glucanases; PR3 and PR4, chitinases; PR5, thaumatin-like proteins; PR6, proteinase inhibitors; PR7, endoproteases; PR8, cucumber chitinase III; PR9 peroxidase; PR10 ribonuclease-like; PR11, chitinase V; and PR12, defensin; PR13, thionin; PR14, lipid transfer proteins; and PR15 and PR16, oxalate oxidases (van Loon, 1985). Additionally, many of the single dominant genes (R genes) identified in diverse collections of a given plant species have also been cloned and bred into plants for disease resistance (Presti et al., 2015). The products of the R genes often recognize specific pathogen effectors (Zhou et al., 1997; Boller and Felix, 2009; Hadwiger and Chang, 2015). These genes are efficiently utilized for crop improvement. However, the resistance they provide can be bypassed by mutations in the effector genes of the pathogen (McDowell and Woffenden, 2003).

The non-host resistance that enables plants outside the host range of a given pathogen to resist their “inappropriate” pathogens is probably more durable because there are diverse types of effectors/elicitors and because multiple resistance traits are involved. To account for the multiple effector/plant protein receptor roles in the PAMP/PRR defense model (Boller and Felix, 2009), one must hypothesize a pre-event presence of an abundant gene bank of plant receptor proteins that is broad enough to match all the diverse effectors the plant may confront. This signaling event must also be capable of transmitting the signal to the site for defense gene transcription (Hadwiger, 2015b). The non-host resistance model with chromatin as a receptor offers flexibility to account for many of the multiple interactions between plants and their pathogens. This resistance developed against an “inappropriate” pathogenic fungus, such as a bean pathogen in pea, can rapidly develop within the pea endocarp tissue (Hadwiger, 2015b). Some major receptors targeted by effectors/elicitors released by these fungi may lie directly within the DNA and proteins of pea chromatin (Isaac et al., 2009). There are diverse mechanisms, such as remodeling or altering transcription and enhancing the properties of chromatin (Li et al., 2007), that result in PR gene activation. The multiplicity of DNA conformations or the modifications of the nuclear proteins in plant chromatin have been described (Choi et al., 2001), which include DNA strand breakage, base substitution, helical changes, deletion/point mutations, nuclear protein removal (ubiquitination) and histone modification or elimination, among others (Li et al., 2007; Lagerwerf et al., 2011).

Thus, the objective of the current research was to evaluate the aspects of legume defense simulation by SA (capable of signaling disease resistance in *Arabidopsis*) that may correspond with the induction of non-host resistance by *Fusarium solani* f.sp. *phaseoli* (*Fsph*), an inducer of non-host resistance in pea tissue. This analysis examined the development of reactive oxygen species (ROS) and DNA damage in pea tissue. Subsequently, the resultant SA-related activation of pea PR genes important to plant defense was monitored with DNA probes from pea genes possessing partial homology to those in *Arabidopsis*.

The molecular response between fungal pathogens and plant cells is rapid if the signaling route excludes surface obstacles, such the cuticle layer. The pea endocarp system was selected because the entire surface lacks a cuticle, and the surfaces of epidermal cells uniformly react to fungal inoculum, providing total resistance to non-pathogenic or inappropriate pathogens within 6 h. Additionally, the nuclei within the surface cell layer can be easily stained and monitored for visible changes. Time course increases in ROS and changes in DNA fragmentation can be readily assayed to evaluate their participation in initiating the transcription of PR genes, especially those with protein products such as the defensins that directly suppress growth of pathogenic spores (Almeida et al., 2000). Increases in ROS have reportedly been implicated in increasing DNA damage. DNA damage in the pea host is also associated with the release of fungal DNase, a mitochondrial DNase from *Fsph* (Klosterman et al., 2001), thus suggesting that the effects of overlapping DNA damage help to initiate gene transcription.

Pea PR genes map to multiple chromosomes and often reside in regions that also map as QTLs (Pilet-Nayel et al., 2002). PR genes are ubiquitously present in plant genomes and possess properties that enable them to be selectively expressed in the resistance response. PR genes with strong antifungal properties are potentially major contributors to resistance (Chiang and Hadwiger, 1991; Almeida et al., 2000). It appears that there is an additive effect of multiple PR genes that results in complete non-host resistance. Pea PR genes share partial homology with the PR genes induced by SA in *Arabidopsis* (Sels et al., 2008). The objective of this research was to determine whether the genes activated by SA respond similarly to those induced by other elicitors in pea endocarp tissue. An additional objective was to determine whether there is an associated release of ROS in the early hours following SA treatment.

The SA affinity to DNA, similar to other previously described DNA-specific agents, can cause DNA damage (Neaualt et al., 1996; Yan et al., 2013). More recently, there have been reports of ATP-dependent chromatin remodelers that allow both transcription factors and the general transcription machinery access to DNA. In addition, these remodelers target specific nucleosomes at the edge of nucleosome-free regions, where they regulate specific transcriptional programs. Nucleosome regions have been identified by DNase 1 digestion assays as areas often encompassing unexpressed genes. This somewhat preferential transcription of PR genes gives credence to the observed selective expression of plant defense resulting from general challenges to sensitive chromatin structures. In cells, the double stranded DNA helix is mostly supercoiled and is either under- or overwound (Ma et al., 2013). RNA polymerase II must transcribe through this supercoiled DNA. For transcription to occur, the DNA helix must be opened as the polymerase threads the separated strands through the enzyme. This process generates supercoiling ahead of and behind the polymerase. The upstream torque disrupts the DNA double strand structure and stalls the polymerase, while the release of this torsional stress allows the polymerase to resume transcription.

DNA damage by microbial enzymes that cause double stranded breaks has also been reported (Song and Bent, 2014), and it is likely that this higher level damage is more of a challenge to the plant than the single strand nicking caused by *Fsph* DNase. Interestingly, the abundance of double strand breaks is reduced by plant defense responses, suggesting that the mechanisms for activating DNA repair processes may share some similarity with the induction of PR genes.

Since SA has recently been reported (Bau et al., 2013) to interact with DNA and has the potential to indirectly influence the state of nuclear DNA by its catalytic inhibition of topoisomerase II, it also has the potential to influence nuclear DNA in plant cells. Single-strand nicks within the large genomic DNA of plants do not produce fragments small enough to be easily detected by typical DNA separations. Therefore, a post-extraction processing of the total DNA was employed to detect the DNA damage occurring in the very early hours of fungal–plant interactions that activate temporally associated defense responses within the host and non-host plant responses (Hadwiger and Adams, 1978). We describe an alkaline buffer treatment protocol that separates the DNA strands. This preparation is incorporated into CHEF gel agar-plug-like disks to entrap the bulk of the plant genomic DNA while allowing shorter fragments, now single stranded, to be released in adjacent alkaline buffer and quantified (Choi et al., 2001). Thus, the extent of host DNA damage could be based on the amount of fragments released. We have observed that SA can target and fragment pea DNA. There was a release of ROS that may additionally serve as a signaling component. The SA-generated signals appeared inefficient at activating the secondary metabolism required to produce maximal amounts of pisatin. The transcription response to the SA and fungal challenges was measured with PCR measurements of alterations in the expression of the selected PR genes.

## Materials and Methods

### Plant Material

Pea endocarp tissue was obtained from immature pea pods harvested directly from greenhouse-grown (Samish) peas. The pod halves were separated, and the elicitor treatments were applied to the exposed endocarp tissue.

### Luminol-Based Oxidative Burst Assay

Immature, 2-cm-long pea pods were cut in half. For each sample, one piece (∼1 cm in diameter) was immersed in deionized water in a single well of a white 24-well microplate (PerkinElmer). After an overnight incubation, the solution in the well was exchanged with assay solution containing 100 μM of L-012 (luminol analog; Wako) and 20 μg/ml of horseradish peroxidase (Sigma–Aldrich), with or without SA. The luminescence from each well was measured using an EnSpire multimode plate reader (PerkinElmer).

### Fungal Material

The bean pathogen *F. solani* f.sp. *phaseoli*, Snyder and Hansen (*Fsph*) (ATCC no. 38135) was donated from the Doug Burke lab, and the pea pathogen *F. solani* f.sp. *pisi* (*Fspi*) was obtained from Lindon Porter, IAREC, Prosser, WA.

### Plant Nucleic Acid Extraction and Quantitation

Plant tissue was extensively ground in a mortar with liquid N_2_, glass beads, and the nucleic acids were extracted in buffer no. 1 [5 M sodium perchlorate, 0.5 M Tris base, 2.5% (w/v) SDS, 0.05% (w/v) NaCl, 0.05 M EDTA]. DNA/RNA were precipitated with 95% (v/v) ethanol, and the pellet was redissolved in water, subsequently extracted with chloroform/phenol, and redissolved in water. The RNA was precipitated from the extract by treating the solution with 2 M lithium chloride. The RNA pellet and the ethanol-precipitated DNA from the supernatant were quantitated in a spectrophotometer at 260 nm. Aliquots of the total DNA were electrophoretically separated on standard 1% (w/v) agarose gels. In addition, 30 μg of the total of each treatment was incorporated into 1 ml of 1% (w/v) CHEF gel (in a 1.5 diameter well) under alkaline conditions to cause DNA strand separation. The solidified gel disk was overlaid with 1 ml of alkaline buffer (30 ml 1 N NaOH and 8 ml 0.5 M EDTA/L) and rotated for 48 h. The DNA fragments eluted into the overlay were precipitated and separated on standard agarose gels. All of the treatments were repeated with similar results.

### Cytological Detection of Treatment- induced Nuclear Changes in Pea Endocarp Tissue

Changes in the nuclear structure and nuclei diameter were imaged with a fluorescent microscope following staining with the DNA-specific dye, DAPI. Subsequently, the diameters of the nuclei from the digital images were uniformly amplified by photocopying, and 45 nuclei from each treatment were manually measured.

### Quantitative Real-time RT-PCR (qRT-PCR)

The procedures for the total RNA isolation and purification were performed as described above. The total RNA was subjected to qRT-PCR using a CFX96 Touch Real-Time PCR Detection System (Bio-Rad Laboratories, Inc.) The primers used were described in our previous research (Hadwiger and Tanaka, 2015).

## Results and Discussion

### Effect of SA on the Production of ROS in Pea Endocarp Tissue

**FIGURE 1:**
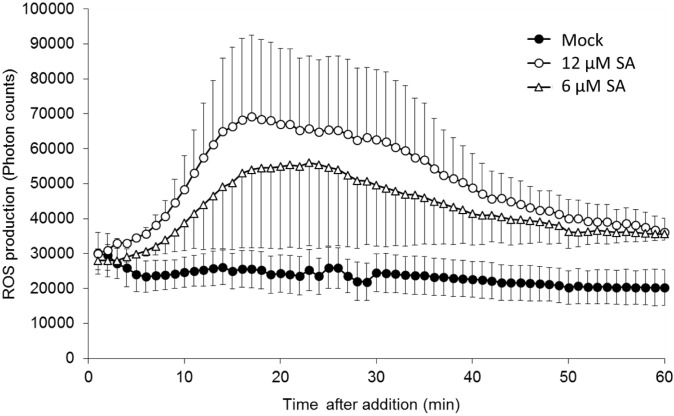
**Salicylic acid (SA)-induced reactive oxygen species (ROS) accumulation in pea endocarps**. Half-cut pea endocarps were treated without or with 6 or 12 μM of SA. Treatment with water (Mock) was used as a negative control. The data show the photon counts in 1.0 s at each time point with SE (*n* = 4).

An “oxidative burst” is the rapid release of ROS from stressed plant cells that develops when they come into contact with different pathogens (Grant and Loake, 2000). To detect this early ROS response in pea, a luminol-based oxidative burst assay was performed. As shown in **Figure 1**, SA treatment induced an oxidative burst with a peak at ∼20 min, whereas water treatment (mock) did not induce an oxidative burst. This method is quite robust and sensitively captures the dynamic changes in ROS production at an early time point in the pea endocarp.

Salicylic acid applied 20 min prior to the inoculum at certain concentrations significantly reduced the linear growth of the true pea pathogen *F. solani* f.sp. *pisi* (*Fspi*) on the endocarp surface. The gradation of action relative to the SA concentration was reproducible over two extensive trials. One of the trials is presented in **Table 1**, while the other is not shown. The growth of the pea pathogen *Fspi* on the pea endocarp surface is less than that on water-treated tissue.

**Table 1:** Effect of salicylic acid (SA) treatments on the subsequent 24 h growth of *Fspi* on pea endocarp tissue.

Treatment^a^	Concentration	Linear growth of *Fspi*^b^
SA	100 μM	2.29 ± 1.29
SA	50 μM	2.53 ± 1.50
SA	25 μM	1.04 ± 0.94
SA	12 μM	0.33 ± 0.33
SA	6 μM	1.29 ± 1.10
SA	3 μM	0.79 ± 0.78
SA	1.6 μM	1.19 ± 0.49
SA	0.7 μM	0.85 ± 0.75
SA	0.3 μM	2.03 ± 0.73
SA	0.15 μM	0.10 ± 0.10
SA	0.07 μM	0.62 ± 0.62
SA	0.03 μM	1.41 ± 0.41
Water 1	–	1.83 ± 1.50
Water 2	–	1.73 ± 0.43

^a^*The endocarp inner tissues of immature pea pod halves (2 cm) were treated with 25 μl of the indicated treatments. After 20 min, 10 μl of an *Fspi* suspension (6.7 × 10^5^ spores/ml) was applied to each pod half. The two water treatments used were duplicate*.

^b^*The growth of 10 individual cotton blue stained spores per treatment was recorded after 24 h. Numbers indicate the multiples of the length of a *45*-micron macroconidia*.

**FIGURE 2:**
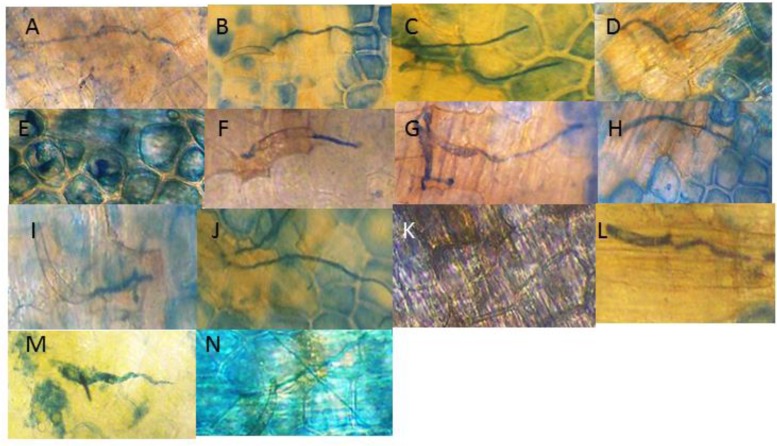
**Visual effect of SA treatments of pea endocarp tissue on the 24 h growth of the true pea pathogen *Fspi***. The images are representative of the calculated growth of the SA treatments (indicated in **Table 1**): **(A)** H_2_0; **(B)** 100 μM SA; **(C)** 50 μM SA; **(D)** 25 μM SA; **(E)** 12 μM SA; **(F)** 6 μM SA; **(G)** 3 μM SA; **(H)** 1.6 μM SA; **(I)** 0.7 μM SA; **(J)** 0.3 μM SA; **(K)** 0.15 μM SA; **(L)** 0.07 μM SA; **(M)** 0.03 μM SA; and **(N)** 0.015 μM SA. The action of SA for causing DNA damage and enhancing resistance appears variable in regions of the SA gradient. This variability may also correspond with its differing resistance action.

Cytological readings (**Table 1**) of the fungal growth began to demonstrate measurable inhibition after 24 h (**Figure 2**). An SA dilution series treatment down to the 0.03 μM showed suppressive effects. The characteristic changes in the background hypersensitivity discoloration of the adjacent pea cells suggest that there was a plant-based change in the suppressive effect. Nearly complete and optimal resistance occurred close to the 0.15 μM SA concentration.

### Effect of SA Concentrations on the *In vitro* Growth of the Pea Pathogen *Fspi*

Salicylic acid had no significant direct effect on *in vitro Fspi* growth in liquid media. The microscopic examination of growth after 24 h in Vogel’s media indicated that the *Fspi* spores germinated and grew uniformly at the concentrations used in **Figure 2** (data not shown).

### SA Induced Changes at the DNA/Nuclear Level

**FIGURE 3:**
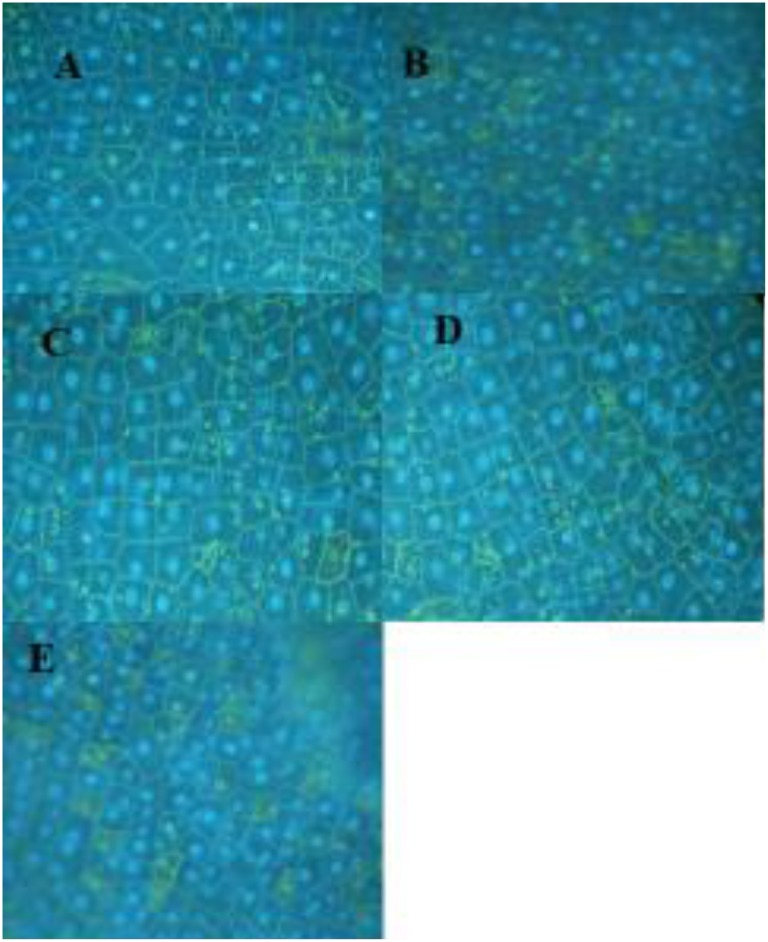
**Pea nuclei treated for 3 h with varying levels of SA**. **(A)** Water-treated control; **(B)** 1 mM SA; **(C)** 0.5 mM SA; **(D)** 0.125 mM SA; and **(E)** 0.06 mM. Nuclei were stained with DAPI.

Following the report that SA has an affinity for DNA (Neaualt et al., 1996), it was of interest to examine changes in pea DNA damage in the nucleus and elsewhere within the pea cells. SA applied to the cuticle-free surface of the pea endocarp tissue rapidly caused cytologically detectable changes in the plant nuclei (**Figure 3** and **Table 2**). These changes were related to the SA concentration and the duration of the SA exposure.

**Table 2:** Diameter of nuclei visible in the endocarp surface following treatment with SA dilutions for 30 min.

Treatment	Concentration applied	Diameter^a^ Average (μm) of 30 nuclei
Water	–	10.000
SA	100 μM	11.792
SA	50 μM	12.669
SA	25 μM	13.774
SA	12.5 μM	9.630
SA	6.25 μM	10.014
SA	3.12 μM	8.738
SA	1.56 μM	11.522
SA	0.78 μM	8.534

^a^*Nuclei were stained for 5 min with DAPI, and the unfixed tissue was imaged under UV light using a fluorescence microscope. Digital images were uniformly printed on full pages, and 30 nuclei were physically measured; the diameters are compared to that of the water-treated control values and standardized to the 10 micron diameter typically observed in electron micrographs*.

**FIGURE 4:**
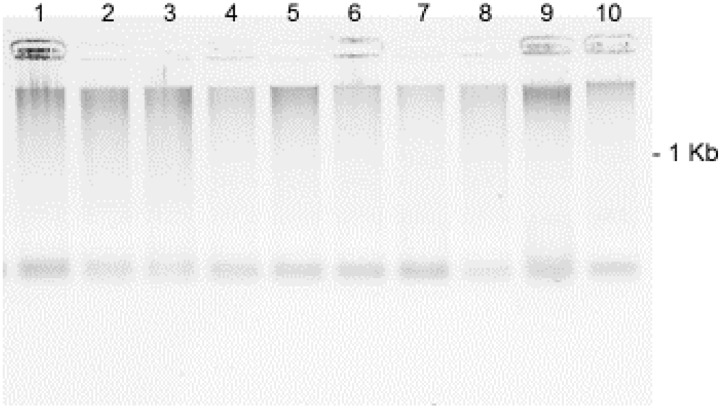
**Effect of SA concentrations on the fragmentation of pea DNA 50 min after the SA treatments: Lane 1 = 100 μM; Lane 2 = 50 μM; Lane 3 = 25 μM; Lane 4 = 12.5 μM; Lane 5 = 6.75 μM; Lane 6 = 3.1 μM; Lane 7 = 1.5 μM; Lane 8 = 0.78 μM; Lane 9 = *Fsph* (5 × 10^6^ spores/ml); and Lane 10 = water-treated control**. The DNA samples loaded onto the gel were aliquots of fragments eluted from 20 μg of total genomic pea DNA (encumbered 48 h in CHEF gel disks) under alkaline conditions (see Materials and Methods). Images represent inverted images of ethidium bromide stained gels.

DNA fragmentation appeared rapidly (50 min post treatment) and variably with the range of SA treatment concentrations and was generally consistent throughout multiple experiments (**Figure 4**). Fragmentation was more intense for the treatment with 100 to 6.75 μM SA and for tissues treated with *Fsph* spores. The specific mechanistic impact of SA on the pea DNA responsible for initiating chromatin transcription is not known for either pea or animal tissues. Maximal transcription of PR genes may depend on a “perfect storm” of conditions and the fragility of chromosomal regions adjacent to the promoter and open reading frame of the gene. Regions of dispersed pea chromatin that are also regions of intense transcription have been detected by electron microscopy (Hadwiger, 2015a) as resistance is developing. Genes within eukaryotic tissues can possess the requisite transcription complex with the proper transcription factors in place and still be silent or stalled (Li et al., 2007). We suggest that there may be stalled PR genes that are activated following major DNA or chromatin structural changes caused by the non-specific SA insults within the adjacent regions.

**FIGURE 5:**
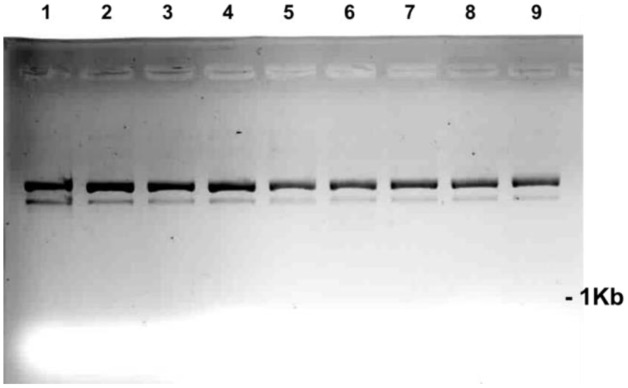
**The direct effect of an array of SA concentrations on the migration of pART7F plasmid DNA**. The plasmid DNA (0.2 μg in 1 μl) was incubated for 1 h in 5 μl of the SA concentrations in the numbered wells: 1 = 1000 μM; 2 = 100 μM; 3 = 50 μM; 4 = 25 μM; 5 = 12.5 μM; 6 = 6.25 μM; 7 = 3.1 μM; and 8 = 1.5 μM. Well 9 contained only plasmid.

The reported interaction of SA and DNA did not cause major changes to plasmid DNA (**Figure 5**). There were detectable, faster migrating DNA molecules generated at the highest SA concentrations. How these minor changes would reflect on the structure of DNA incorporated into pea chromatin is not known. This result may indicate that the DNA fragmentation caused by SA in living tissue could involve additional components.

### Effect of SA on Expression of Pea PR Genes

**FIGURE 6:**
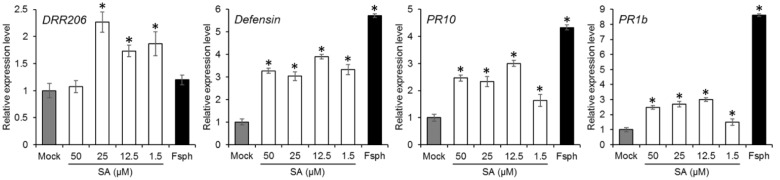
**The effect of SA treatment on pea PR gene expression**. Pea endocarp tissues were treated for 50 min with water (Mock); 50, 25, 12.5, or 1.5 μM of SA; or 5 × 10^6^ spores/ml of fungal spores (*Fsph*). The tissues were then subjected to qRT-PCR analysis in order to measure the transcriptional responses of the pea PR genes. The data were normalized by the reference gene ubiquitin and converted to a value relative to that of the mock treatment. Histograms represent the means with SE in three replicated experiments.

Induction of PR genes is correlated with the activation of plant defense. We measured the transcriptional induction of the pea PR genes, *DRR206*, *Defensin*, *PR10*, and *PR1b* in the presence of SA. The results indicate that the expression of the PR genes induced by SA took place mostly at concentrations between 1.5 and 50 μM (**Figure 6**). The induction levels were comparable to those caused by *Fsph*.

### Elicitation of Pisatin

The elicitation of pisatin, a phytoalexin, serves to indicate the activity of a series of secondary metabolism enzymes from phenylalanine through phenylpropanoid structures to isoflavonoid and other phenolics, many of which have fungal-suppressive properties (Bailey and Mansfield, 1982). Pisatin accumulation is often associated with the induction of immunity in peas (Hadwiger, 2008). The data in **Table 3**, with a high SA concentration range (15–1000 μM), and in **Table 4**, with a lower range (0.7–100 μM), recorded at 24 h indicate detectible levels of SA-induced pisatin. The response with both ranges indicates a much lower pisatin accumulation than that induced by the intact microconidia of *Fsph* during the authentic non-host resistance response. The 1.5 μM SA treatment optimally induced pisatin. However, this value is much lower than the level induced by spores. This result suggests that SA is not a major elicitor of this secondary metabolism route of defense responses in pea at this or higher concentrations of the SA elicitor (**Table 4**).

**Table 3:** The effect of a high concentration range of SA on the production of pisatin in pea endocarps.

Treatment^a^	Concentration	Pisatin^b^ (μg/g fw)
Water	–	0.0 ± 0.0
SA	1000 μM	7.7 ± 0.02
SA	500 μM	5.2 ± 0.59
SA	250 μM	6.6 ± 2.5
SA	125 μM	5.8 ± 2.3
SA	62 μM	2.6 ± 2.6
SA	31 μM	4.9 ± 0.2
SA	15 μM	13.6 ± 4.5
*Fsph*	1 × 10^6^ spores/ml	113.1 ± 20.2

^a^*Treatments (25 μl) were applied to pea pod halves (∼250 mg fresh weight) with the indicated concentrations and subsequently distributed on the surface with a glass rod. Pods were retained in high humidity for 24 h*.

^b^*Pisatin was extracted from pea tissue with hexanes. The hexanes were removed by volatilization, and the pisatin-containing residue was extracted with 95% ethanol and quantified at 309 nm*.

**Table 4:** Effect of a lower concentration range of SA on the 24 h production of pisatin in pea endocarp tissue.

Treatment	Concentration	Pisatin (μg/g fw)
SA	100 μM	3.1 ± 0.8
SA	50 μM	2.0 ± 1.4
SA	25 μM	2.6 ± 0.6
SA	12.5 μM	3.3 ± 0.5
SA	6.2 μM	4.1 ± 1.3
SA	3.1 μM	7.5 ± 1.1
SA	1.5 μM	19.3 ± 4.4
SA	0.7 μM	8.3 ± 3.4
*Fsph*	2.4 × 10^6^ spores/ml	145 ± 3.0

*Legend is the same as that of **Table 3***.

### SA Signal: Complete or Additive Effect on Resistance

The low-level effect of SA on phytoalexin synthesis indicates a departure from the mechanisms of other signals for non-host resistance in pea. However, SA is capable of inducing a response that suppresses the true pathogen of pea and approaches total resistance. The following assay of pisatin production indicates that its effect can be additive to that induced by *Fsph*, a bean pathogen.

The pisatin levels (**Table 5**) indicate a marginal increase in synthesis enhanced in the presence of both *Fsph* and specific SA concentrations. Because of the low strength of the SA-induced pisatin increase, it is likely that the modeling effect of SA on chromatin differs in approach or substance from the DNA single strand cleavage generated by *Fsph* DNase (Klosterman, et al., 2001).

**Table 5:** Assessment of SA additivity to the synthesis of *Fsph*-induced pisatin in pea endocarp tissue after 24 h.

Treatment and molarity^a^	Pisatin (μg/g fw)
Water	0.0
Water + *Fsph* spores	121.3
SA 100 μM	2.3
SA 100 μM + *Fsph* spores	198.8
SA 6.7 μM	5.9
SA 6.7 μM + *Fsph* spores	195.2
SA 3.1 μM	0.7
SA 3.1 μM + *Fsph* spores	174.8
SA 1.5 μM	0.0
SA 1.5 μM + *Fsph* spores	206.0
SA 0.7 μM	0.02
SA 0.7 μM + *Fsph* spores	218.3

^a^*The indicated treatments were applied (25 μl) to the endocarp layer of each pea pod half with, when indicated, 5 μl of *Fsph* spores (1 × 10^7^/ml). Pisatin was extracted (at 24 h) in hexane. The hexane free residue was dissolved in 95% ethanol and quantified at UV_309_*.

The enzymatic action of DNase has also been implicated in initiating the transcription of plant defense genes by directly altering nuclear chromatin via single DNA strand nicking. The resultant DNA damage has to be subtle enough to alter chromatin structure in a manner that benefits the pathogen and yet does not initiate processes that could cause immediate cell death (Choi et al., 2001). DNA damage by microbial enzymes that cause double stranded breaks has also been reported (Song and Bent, 2014), and it is likely that this higher level damage is more of a challenge to the plant than the single strand nicking caused by *Fsph* DNase. Interestingly, the abundance of double strand breaks is reduced by plant defense responses, suggesting that the mechanisms for activating DNA repair processes may share some similarity with the induction of PR genes (Gasser et al., 2005; Yan et al., 2013).

### Origin of the SA Signal

**FIGURE 7:**
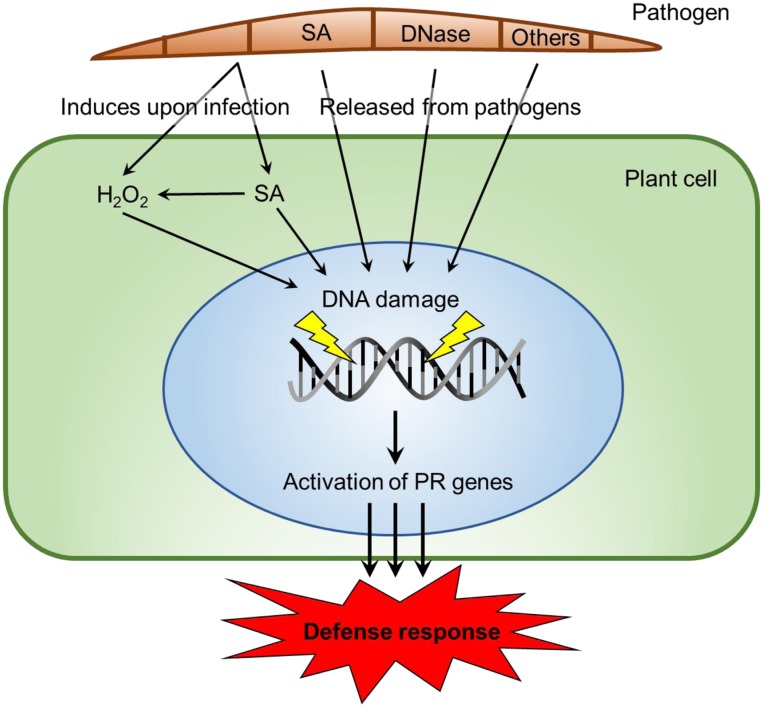
**Possible origins of SA signals and mechanisms of DNA damage in host parasite interactions**. SA is produced by demethylation of Methyl-SA or by biosynthesis from cinamate or isochorismate in plants (Raskin, 1992). Alternatively, SA is produced via chorismate in bacteria and fungi (Harper and Hamilton, 1988). Fungi have DNases released (Hadwiger and Polashock, 2013) or other biotic agents. Those agents elicit DNA damage or conformational changes (Hartney et al., 2007), via ROS production at one stage (Driessens et al., 2009; Tanaka and Hadwiger, 2017). We propose that DNA-damaging elicitors can act singly or with assistance at differing DNA sites and may potentially activate PR genes. The elicitor insults to chromatin structure have the ability to restart stalled RNA polymerase complexes.

Some current possibilities for the origin, presence, and availability of the SA signal are described in **Figure 7**. SA is synthesized by bacteria and some fungi (Harper and Hamilton, 1988). SA and methyl-SA can be found in the plant tissue prior to infection and be stored as a byproduct (Maeda and Dudareva, 2012). Hydrogen peroxide is generated in inoculated plant tissue (Coquoz et al., 1988) as tissue damage occurs. In tomatoes, the wound hormone systemin is also produced (Orozco-Cardenas and Ryan, 1999). Hydrogen peroxide can also generate increases in SA. Plants biosynthesize SA using the phenylalanine/cinnamic acid pathway or alternately via benzoic acid (Chen et al., 2009). Both hydrogen peroxide and SA are capable of damaging host DNA. Fungal DNase can directly cleave a single DNA strand. The gene for this potent elicitor has been identified in all fungi whose DNA has been sequenced (Hadwiger and Polashock, 2013). All the DNase proteins are translated with a “signalP peptide” that enables proteins to pass through membranes. Many other eliciting components may be released from fungi, such as the chitosan heptamer that is released from the fungal cell wall (Kendra et al., 1989).

Since SA has recently been reported (Bau et al., 2013) to interact with DNA and has the potential to indirectly influence the state of nuclear DNA by its catalytic inhibition of topoisomerase II, it has the potential to also influence nuclear DNA in plant cells. Single-strand nicks may have previously escaped observance due to their low abundance within the total, large genomic DNA yields from plants. The alkaline processing and agarose trapping of the total DNA enabled the detection of released DNA fragments (see Materials and Methods) that occur in the very early hours of the inductive treatments (**Figure 5**). This fragmentation was temporally associated with the initiation of defense responses. The sheer presence of the SA association with host DNA is not likely to result in DNA alterations without the presence of a contributing factor such as the direct *in vitro* association of various SA concentrations.

### Mechanisms for Regulating PR Gene Expression

Pea PR genes map to multiple chromosomes and often reside in regions that also map as QTLs (Pilet-Nayel et al., 2002). PR genes are ubiquitously present in plant genomes and possess antifungal properties, and therefore, they are potentially the major contributors (Chiang and Hadwiger, 1991; Almeida et al., 2000) to disease resistance. It appears that it is the additive effect of multiple PR genes that develops the complete non-host resistance. Additionally, the pea PR genes analyzed share some homology with the PR genes induced by SA in *Arabidopsis* (Sels et al., 2008).

### Role of Chromatin

Gene expression is initiated within chromatin, the site of transcription. The DNA transcription within the region of defense genes can be up regulated or down regulated depending on the associated chromatin structure. Chromatin is a complex of proteins and DNA packed into nucleosomes (Li et al., 2007). The DNA of a particular gene can be accessed by transcription complexes following the alteration of DNA supercoiling or modifications of the relevant proteins. In pea endocarp tissue, PR gene activation is influenced by DNA alterations, by ubiquitination/histone modifications and by a reduction in the architectural transcription factor HMG A (Klosterman et al., 2003; Isaac et al., 2009).

DNA damage can result in the stalling of elongating RNA polymerase II (Lagerwerf et al., 2011) and the attraction of chromatin remodelers to damaged sites. Chromatin modifications function by either disrupting chromatin contacts or affecting the recruitment of non-histone proteins to chromatin (Kouzarides, 2007). Histone modifications can dictate the higher-order chromatin structure in which DNA is packaged, affecting many biological processes. Chromatin structure itself imposes obstacles on all aspects of transcription that are mediated by RNA polymerase II (Li et al., 2007). The resultant chromatin regulation affects the binding of transcription factors and the initiation and elongation steps of transcription. SA reportedly has an affinity for DNA, and similar to other previously described DNA-specific agents, it can cause DNA damage (Neaualt et al., 1996; Yan et al., 2013). The somewhat preferential transcription of PR genes gives credence to the observed selective expression of plant defense resulting from general challenges to sensitive chromatin structures. For transcription to occur, the DNA helix must be opened as the polymerase threads the separated strands through the enzyme (Ma et al., 2013). The upstream torque disrupts the DNA double strand structure and stalls the polymerase, while release of this torsional stress allows the polymerase to resume transcription.

### Other DNA Specific Elicitors

Chitosan, a fungal-derived elicitor of PR genes, can compete with histones for sites on DNA and can insert itself into the minor groove of DNA. Fungal DNase (*Fsph* DNase), a second major elicitor of PR gene expression, causes single strand cleavage in double stranded DNA, enabling the release of tension within the DNA helical structures (Gerhold et al., 1993). Multiple regulatory substances are released from fungal spores following inoculation on their respective host tissues. Of current interest are the proteins that exit the fungal cell via their SignalP sequences (Ramachandran et al., 2016). The functional properties of defense proteins (described above) can range from those that specialize in digesting the cell wall barriers to those that are metabolic enzymes or proteins with unknown function. The regulatory functions affected within the host tissue may also occur either through receptors and subsequent signal cascades that target transcription factors or as direct insults to the organization of sites within chromatin that result in increases in transcription of host genes (Hartney et al., 2007). Although the signaling of compounds of fungal origin can be shown to specifically complex with membrane proteins and modulate the plant’s response, the resulting signaling cascade to the site of defense gene transcription is less well understood than in many other host/parasite interactions (Presti et al., 2015). High throughput genomic analyses may be able to detect a multiplicity of potential effectors with the potential to traverse the host-parasite barrier. If so, future research should focus on determining which effectors display potential to play a major role in the processes that result in the development of resistance or enable a susceptible response.

The biotic and abiotic elicitors of PR genes, such as the single strand cleaving DNase elicitor from *Fsph*, require a SignalP sequence (Hadwiger and Polashock, 2013). Since homologous fungal genes for DNase production are present in all of the fungal genomes sequenced to date, this chromatin modeling may be implicated in similar signaling in many other plant/fungal interactions (Hadwiger and Polashock, 2013). DNase activity has also been shown to be released from spores of rust (*Puccinia striiformis*), *Verticillium dahliae*, *Colletotrichum coccodes* and yeast cells (Hadwiger and Polashock, 2013). The universality of this enzyme suggests that it could be a general elicitor of the non-host resistance response, protecting plants from pathogens known to be out of their host range. DNase enzymatic action has also been implicated in the initiation of plant defense gene transcription by directly altering nuclear chromatin via single DNA strand nicking. The resultant DNA damage must be subtle enough to alter the chromatin structure for the benefit of the pathogen but not initiate processes that could cause immediate cell death.

## Conclusion

Salicylic acid is a signal that induces a defense response in *Arabidopsis* and some other plant species (Heil and Bostock, 2002). The data presented also indicate that SA can activate a defense response in pea that is associated with the activation of pea PR genes possessing partial homology with those in *Arabidopsis*. Similar to the signals that activate genes in pea, there is a surge in ROS release within 40 min and temporal DNA damage within 90 min that is detectible in pea DNA fragmentation, in addition to changes in its nuclear appearance and diameter. Although the phytoalexin accumulation is only slightly affected by SA, the effects on the transcription of pea PR genes via DNA damage and distortion may indicate that a signaling route targeting host DNA implicates a different type of chromatin remodeling or transcription initiation. SA may also complement the transcriptional enhancing effect directly on DNA by utilizing a membrane receptor and a subsequent cascade of events that alter the transcription complex by transcription factor attraction or modification. This report shows that ROS that are capable of DNA modification are released. There were nuclear and DNA alterations similar to the changes in other systems that have been associated with enhanced transcription. Additionally, these changes are temporal in the phase that is crucial for the activation of PR genes and non-host resistance in pea endocarp tissue.

## Author Contributions

LH conceived and designed the experiments, LH and KT conducted the experiment, data analysis, presentation, and wrote the manuscript.

## Conflict of Interest Statement

The authors declare that the research was conducted in the absence of any commercial or financial relationships that could be construed as a potential conflict of interest.
